# VDJML: a file format with tools for capturing the results of inferring immune receptor rearrangements

**DOI:** 10.1186/s12859-016-1214-3

**Published:** 2016-10-06

**Authors:** Inimary T. Toby, Mikhail K. Levin, Edward A. Salinas, Scott Christley, Sanchita Bhattacharya, Felix Breden, Adam Buntzman, Brian Corrie, John Fonner, Namita T. Gupta, Uri Hershberg, Nishanth Marthandan, Aaron Rosenfeld, William Rounds, Florian Rubelt, Walter Scarborough, Jamie K. Scott, Mohamed Uduman, Jason A. Vander Heiden, Richard H. Scheuermann, Nancy Monson, Steven H. Kleinstein, Lindsay G. Cowell

**Affiliations:** 1 Department of Clinical Sciences, UT Southwestern Medical Center, 5323 Harry Hines Boulevard, Dallas, TX 75390-9066 USA; 2 Bank of America Corporate Center, 100 North Tryon Street, Charlotte, NC 28202 USA; 3 Broad Institute, 75 Ames Street, Cambridge, MA 02142 USA; 4 Institute for Computational Health Sciences, University of California San Francisco, Mission Hall, 550 16th Street, 4th Floor, Box 0110, San Francisco, CA 94158 USA; 5 Department of Biological Sciences and The IRMACS Centre, Simon Fraser University, 8888 University Drive, Burnaby, V5A 1S6 British Columbia Canada; 6 Department of Immunobiology, University of Arizona School of Medicine, 1656 E. Mabel Street, P.O. Box 245221, Tucson, AZ 85724-5221 USA; 7 New Zealand eScience Infrastructure, University of Auckland, Level 10, 49 Symonds Street, Auckland, New Zealand; 8 Texas Advanced Computing Center, Research Office Complex 1.101, J.J. Pickle Research Campus, Building 196, 10100 Burnet Road (R8700), Austin, TX 78758-4497 USA; 9 Interdepartmental Program in Computational Biology and Bioinformatics, Yale University, 300 George Street, Suite 505, New Haven, CT 06511 USA; 10 School of Biomedical Engineering, Science and Health Systems and Department of Microbiology and Immunology, College of Medicine, Drexel University, 3141 Chestnut Street, Philadelphia, PA 19104 USA; 11 The IRMACS Centre (ASB 10905), Simon Fraser University, 8888 University Drive, Burnaby, BC V5A 1S6 Canada; 12 School of Biomedical Engineering, Science and Health Systems, Drexel University, 3141 Chestnut Street, Philadelphia, PA 19104 USA; 13 Department of Neurology and Neurotherapeutics, UT Southwestern Medical Center, 5323 Harry Hines Boulevard, Dallas, TX 75390-9036 USA; 14 Stanford University School of Medicine, 279 Campus Drive, Stanford, CA 94305-5101 USA; 15 Department of Molecular Biology and Biochemistry and Faculty of Health Sciences, Simon Fraser University, Blusson Hall, Room 11300, 8888 University Drive, Burnaby, BC V5A 1S6 Canada; 16 Department of Pathology, Yale School of Medicine, 300 George Street, Suite 505, New Haven, CT 06511 USA; 17 J. Craig Venter Institute, 4120 Capricorn Lane, La Jolla, CA 92037 USA; 18 Department of Pathology, University of California, San Diego, 9500 Gilman Drive, La Jolla, CA 92093 USA; 19 Division of Vaccine Discovery, La Jolla Institute for Allergy and Immunology, 9420 Athena Circle, La Jolla, CA 92037 USA; 20 Department of Immunobiology, Yale School of Medicine, New Haven, CT USA

**Keywords:** Repertoire profiling, Immune repertoire, Antigen receptor repertoire, Data standards, Data sharing, Python, C++, XML

## Abstract

**Background:**

The genes that produce antibodies and the immune receptors expressed on lymphocytes are not germline encoded; rather, they are somatically generated in each developing lymphocyte by a process called V(D)J recombination, which assembles specific, independent gene segments into mature composite genes. The full set of composite genes in an individual at a single point in time is referred to as the immune repertoire. V(D)J recombination is the distinguishing feature of adaptive immunity and enables effective immune responses against an essentially infinite array of antigens. Characterization of immune repertoires is critical in both basic research and clinical contexts. Recent technological advances in repertoire profiling via high-throughput sequencing have resulted in an explosion of research activity in the field. This has been accompanied by a proliferation of software tools for analysis of repertoire sequencing data. Despite the widespread use of immune repertoire profiling and analysis software, there is currently no standardized format for output files from V(D)J analysis. Researchers utilize software such as IgBLAST and IMGT/High V-QUEST to perform V(D)J analysis and infer the structure of germline rearrangements. However, each of these software tools produces results in a different file format, and can annotate the same result using different labels. These differences make it challenging for users to perform additional downstream analyses.

**Results:**

To help address this problem, we propose a standardized file format for representing V(D)J analysis results. The proposed format, VDJML, provides a common standardized format for different V(D)J analysis applications to facilitate downstream processing of the results in an application-agnostic manner. The VDJML file format specification is accompanied by a support library, written in C++ and Python, for reading and writing the VDJML file format.

**Conclusions:**

The VDJML suite will allow users to streamline their V(D)J analysis and facilitate the sharing of scientific knowledge within the community. The VDJML suite and documentation are available from https://vdjserver.org/vdjml/. We welcome participation from the community in developing the file format standard, as well as code contributions.

## Background

The genes that encode antibodies (Ab) and the immune receptors expressed on B and T lymphocytes are not germline encoded; rather, they are somatically generated in each developing lymphocyte by a process called V(D)J recombination, which assembles specific, independent germline gene segments into mature, composite genes [1]. Seven types of genes are assembled by V(D)J recombination, and, for each one, there are two or three sets of gene segments: Variable (V) and Joining (J) gene segments are present in all seven, and Diversity (D) gene segments are present in three. During V(D)J recombination, one gene segment of each type is selected, essentially at random, and the selected segments are joined to form a rearranged gene [2]. A diverse repertoire of genes is created as a result of the varied combinations of gene segments. In addition to this combinatorial diversity, there is junctional diversity as a result of imprecise joining: the sequence at the junction of two joined gene segments is unique due to enzymatic processes that act on the gene segment ends (e.g., hairpin opening and exonucleolytic removal) and add non-templated nucleotides into the junctions [2]. In B lymphocytes, the rearranged genes are further diversified through gene conversion (e.g., in chickens and rabbits) [3] or somatic hypermutation (e.g., in mice and humans) [4]. As a result of these processes, each individual has millions of unique Ab and immune receptor genes [5, 6].

V(D)J recombination is the distinguishing feature of adaptive immunity and, through the creation of a diverse immune receptor repertoire, enables the mounting of an effective immune response against an essentially infinite array of antigens, such as those derived from pathogens or tumors. It also has the potential to generate autoimmune responses. Thus, the characterization of adaptive immune receptor repertoires is critical in both basic research and clinical contexts, as well as in the development of pharmaceuticals. Recent application of high-throughput sequencing allows description of the immune response in exquisite detail and has resulted in an explosion of research activity in the field [7–10]. This has been accompanied by a proliferation of software tools for analysis of repertoire sequencing data.

Repertoire sequencing typically involves targeted polymerase chain reaction (PCR) or 5′ rapid amplification of cDNA ends (5′ RACE) to amplify rearranged gene sequences followed by sequencing of the PCR product. The initial steps in analysis of the resulting sequencing data are generally the same, regardless of the biological or clinical question being addressed [11]. The first step is preprocessing to prepare reads for analysis. Examples of preprocessing activities include demultiplexing, quality filtering, and error correction. The second common analysis step is inference of rearranged gene sequences via alignment of sequences to reference databases of germline gene segments. The third step is annotation of rearranged gene sequences according to things such as gene segments utilized, location of complementarity determining regions (CDRs), non-templated nucleotides, and, in the case of B lymphocyte-derived sequences, base substitutions, insertions, and deletions resulting from somatic hypermutation. The fourth common step is repertoire characterization, including clone enumeration, determination of repertoire-level distributions (e.g., gene segment usage, CDR3 length), and quantification of diversity and clonality. A large number of software packages have been developed for these analyses, particularly for the second and third steps. At the time of this writing, there are no fewer than 24 packages, each performing at least one of steps 2–4, and there will certainly be many more released in the future [12–35].

Despite the importance and widespread application of immune repertoire profiling via high-throughput sequencing, there are currently no community standards for data recording and exchange. The inputs and outputs for preprocessing software packages are reasonably standardized, as these mainly read and write FASTA/QUAL or FASTQ files. There are, however, differences in the way sequence-level metadata (such as primer matches and UID sequences) are stored, with one mechanism being encoding this information as entity-value pairs directly in the FASTQ format [24]. Software packages for conducting germline alignment all utilize FASTA files, but they write different output formats, and, for all subsequent analysis steps, there are no input/output standards. The most widely used germline alignment packages, IgBLAST and IMGT/High V-QUEST, write distinct mixed content text files that intersperse sequence alignments and tables with data embedded in free text. This creates significant problems for both software developers and users. Software developers must write parsers for multiple different input formats or limit their software to consuming the output of only a single alignment package. Users must reformat data as it moves through an analysis pipeline, which is a time-consuming and error-prone process. This problem is exacerbated by the frequent desire to utilize multiple packages for a single analysis task for comparative purposes. Furthermore, reformatting is not always possible as the formats differ not just in how the data are represented but also in the content.

To address this problem, we have developed VDJML, an XML-based file format for representing the alignments of rearranged gene sequences to germline gene segments and the accompanying set of annotations. VDJML can accommodate rearrangements from both B and T lymphocytes. VDJML is a common, open standard designed to provide both syntactic and semantic interoperability between software packages. The VDJML suite includes a schema for the VDJML format and libVDJML, a C++ library for generation and parsing of VDJML files, with an accompanying package of Python bindings for the library.

While our primary motivation was to provide a common open standard for software development, VDJML has additional benefits. It can serve as the basis for integrative analysis of results from different studies, and it can meet the widely recognized need for data sharing to improve reproducibility and scientific rigor [8, 36].

## Implementation

### Design priorities

VDJML development was initiated as part of the VDJServer project (https://www.vdjserver.org). It is being developed as an open, community standard. Its data structure was established during regular calls among the authors, some of whom are computational biologists developing repertoire analysis software, and some of whom are experimentalists and users of this software. The team includes developers of VDJServer and its software packages (e.g. VDJPipe) (https://bitbucket.org/account/user/vdjserver/projects/VDJS), developers of iReceptor, and developers of the Immcantation framework (http://immcantation.readthedocs.io) [23, 24]. Documentation for VDJML is available from https://vdjserver.org/vdjml/. Source code for the tools is available from the VDJML Bitbucket repository at https://bitbucket.org/vdjserver/vdjml.

We chose XML, the eXtensible Markup Language [37], because it readily accommodates key features of our data type, including:

- The need to add or eliminate data fields over time and to version future extensions of the schema,
- The need for standard VDJML data types along with custom, user-defined data types, and
- Variable cardinality relationships between rearranged gene sequences and their annotations (e.g., a single sequence may have alternative alignments, each with multiple base substitutions).

Additional reasons for selecting XML include the portability of XML documents, existing extensive support for standard definitions, parsing, generation, and versioning, and its widespread acceptance as a standard [38].

We had the following additional design priorities:

- Support for including results from multiple software packages, multiple germline databases, and multiple systems for annotating codon positions in rearranged gene sequences;
- Normalization to minimize data duplication;
- Read/write streamability in that, during reading or writing VDJML files, only the information from a single record needs to be present in memory.

### Schema overview

**Fig. 1:**
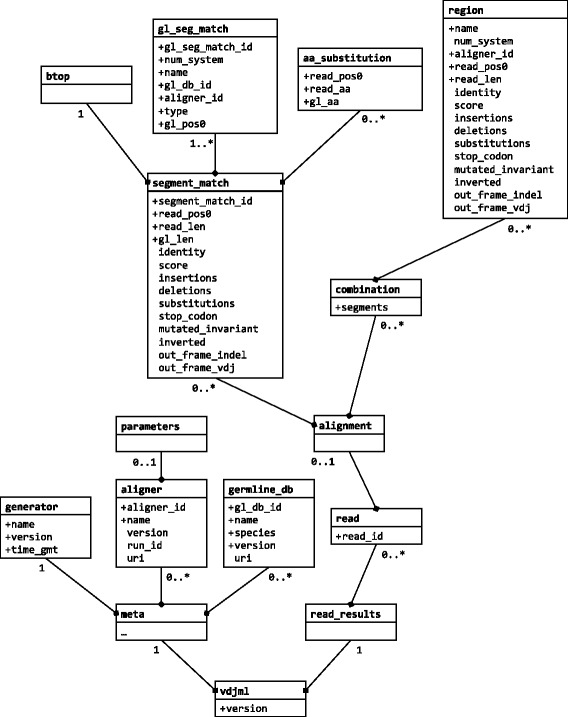
A UML representation of the VDJML schema showing the current scope of VDJML and how the high-level data elements relate to each other. Each *box* corresponds to an element. Attributes are listed within a box. A “+” symbol beside an attribute name indicates that it is required. Labels on edges connecting an element to a child element indicate the number of instances of a child element type that can be included in a VDJML document

Currently, version 1.0 of VDJML has been finalized with a relatively narrow scope that will be extended over time. The current scope does, however, include sufficient information to recreate an alignment. Elements defined by the schema (Fig. 1) belong to the namespace http://vdjserver.org/vdjml/xsd/1/ and are prefixed vdj:. The top element of the schema is vdj:vdjml, which contains the required version attribute.

**Fig. 2:**
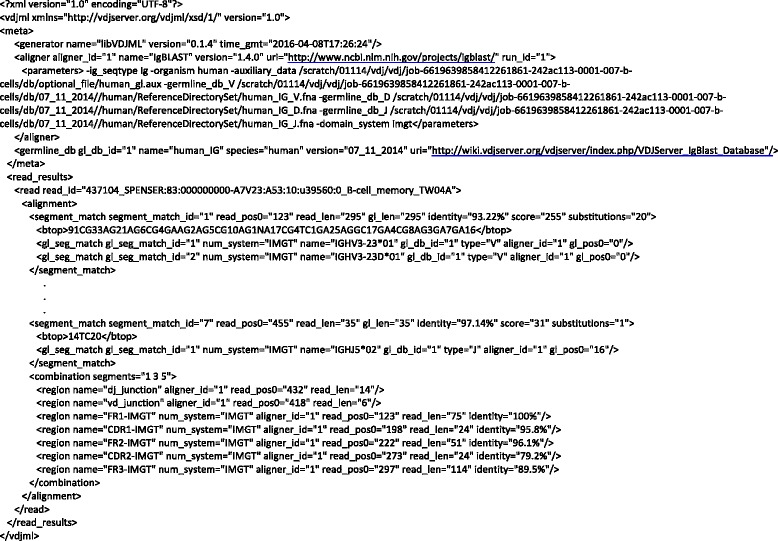
A VDJML file generated on VDJServer. This figure shows the two main parts of a VDJML file, the vdj:meta and vdj:read_results elements. It also shows how information about how the file was generated is recorded in the vdj:meta section. The alignment corresponding to this VDJML file was generated using a local version of IgBLAST. Six of seven vdj:segment_match elements are not shown due to space limitations. These can be seen in Fig. 4

A VDJML file consists of two parts enclosed in the vdj:meta and vdj:read_results elements (Fig. 2). The schema allows user-defined elements and attributes to appear under vdj:meta and vdj:read_results, but these should have namespaces other than vdj.

The vdj:meta element contains general information that may be shared across analysis results (Fig. 2). Its child elements include vdj:generator, vdj:aligner, and vdj:germline_db. The vdj:generator element describes the software that wrote the VDJML file using the required name, version, and time_gmt attributes. The value for the time_gmt attribute is the date and time the file was written in Greenwich Mean Time (GMT). The vdj:aligner element contains information about a software package used to align sequences to a database of germline gene segments, a program that generated all or some of the results in the VDJML document. This element has the required attributes aligner_id and name. The value for aligner_id is a unique identifier that is referenced within child elements of the vdj:read_results element described below. It enables inclusion of results from multiple different aligners for a single sequence in a single VDJML file. vdj:aligner has one child element, vdj:parameters, which can be used to capture information needed to reproduce the run of the alignment software. Figure 2 shows a VDJML file generated on VDJServer using a local installation of IgBLAST. On VDJServer, the parameter element captures the command passed to IgBLAST. The vdj:germline_db element stores information about a germline database used for analysis with the required attributes version, species, name, and gl_db_id. As with aligner_id, the value for gl_db_id is a unique identifier that is utilized with child elements of vdj:read_results to accommodate alignments for a single sequence against multiple germline databases.

### Representation of alignments

Alignment results (alignments plus their annotations) are stored inside the vdj:read_results element as a series of vdj:read elements. Each vdj:read element corresponds to one sequence. The required read_id attribute holds a unique identifier for the sequence, which is the corresponding identifier from the FASTA or FASTQ source file used as input to the alignment software package. The primary child element for vdj:read is vdj:alignment, which captures all of the alignment output for that particular read sequence. It has two child elements: vdj:segment_match and vdj:combination.

The foundation of an alignment is the aligned region of a sequence, the germline gene segments to which the region aligns, and the alignment positions. This information is captured in VDJML using the element vdj:segment_match. This element takes the read sequence as its point of reference and specifies the subsequence that aligns well (or matches) to a particular germline gene segment (or set of gene segments if the alignments are identical). A single vdj:alignment element can have an unlimited number of vdj:segment_match child elements. Thus, this element has segment_match_id as a required attribute. Additional required attributes are read_pos0, read_len, and gl_len, which capture the position in the sequence where the alignment to a particular germline gene segment begins, the number of positions in the sequence that align to the germline gene segment, and the number of positions in the germline gene segment that align, respectively. Optional attributes capture the percentage of positions that match over the aligned region (identity) and the alignment score (score) as defined by the software package generating the alignment. Additional optional attributes (e.g., substitutions) capture more details about the alignment, as described below (Table 1).

**Table 1:** Names and descriptions of optional attributes for segment match and region elements

Attribute	Description
num_system	Designates the numbering system (e.g., Kabat, IMGT) used to number codon positions
identity	Percent of nucleotide sequence identity (e.g., 90 %) between aligned portions of a read sequence and a germline gene segment sequence
score	Alignment score, as defined by the aligner software
insertions	Number of nucleotide insertions in the read sequence relative to the germline sequence
deletions	Number of nucleotide deletions from the read sequence relative to the germline sequence
substitutions	Number of nucleotide substitutions in the read sequence relative to the germline sequence
stop_codon	True if a stop codon is present in the read sequence
mutated_invariant	True if a codon for a conserved amino acid is mutated in the read sequence
inverted	True if the read sequence is a reverse-complement to a germline gene segment
out_frame_indel	True if an insertion or deletion resulted in a frame shift
out_frame_vdj	True if the V(D)J recombination occurred out of frame

Child elements of vdj:segment_match are vdj:btop, vdj:gl_seg_match, and vdj:aa_substitution. The vdj:btop element captures the BLAST “trace-back operations” string. The vdj:gl_seg_match element specifies the germline gene segment in the alignment. It has the unique identifier gl_seq_match_id as a required attribute, because a single vdj:segment_match element can specify alignment to multiple different germline gene segments when those alignments are identical. The required attributes type, name, and gl_pos0 specify the gene segment type (V, D, or J), the gene segment name according to the germline database used, and the first position in the germline gene segment sequence that aligns to the read sequence. Additional required attributes reference the gl_db_id (germline database identifier) and aligner_id (alignment software package identifier) attributes of the vdj:germline_db and vdj:aligner elements in the vdj:meta section of the file described above.

**Fig. 3:**
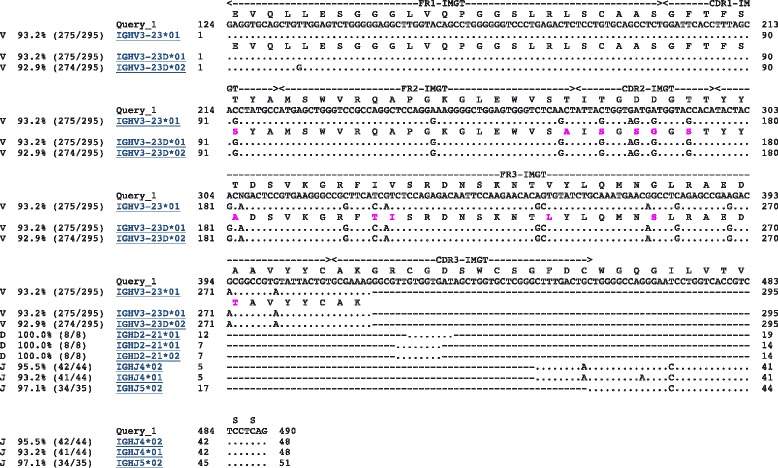
An IgBLAST-generated alignment of an IGH sequence. The sequence was taken from [40]. The standard IgBLAST alignment output is shown

Figure 3 shows a sample alignment generated by IgBLAST for a sequence derived from the IGH locus. Because the example sequence was derived from the IGH locus, we expect alignment to V, D, and J gene segments. Figure 3 shows that the sequence aligns equally well to the V gene segments IGHV3-23*01 and IGHV3-23D*01. It aligns equally well to the D gene segments IGHD2-21*01 and IGHD2-21*02, with two possible alignments to IGHD2-21*01. Finally, the sequence aligns best to the J gene segment IGHJ4*02. There are additional lower scoring alignments to IGHV3-23D*02, IGHJ4*01, and IGHJ5*02.

**Fig. 4:**
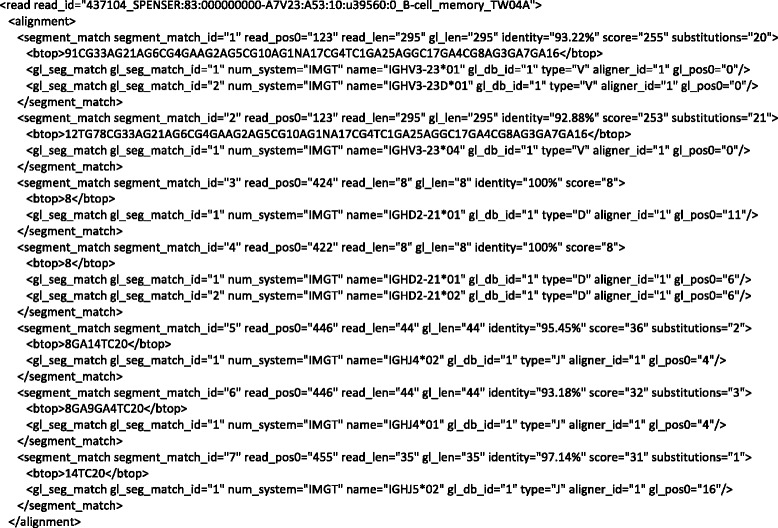
The full vdj:alignment element from the VDJML file shown in Fig. 2. This figure illustrates how the sections of a vdj:alignment element jointly specify a full germline alignment

This information is captured in VDJML using seven vdj:segment_match elements. Figure 2 shows the corresponding VDJML file with some of the vdj:segment_match elements left out for space. Figure 4 shows the full vdj:alignment element with all vdj:segment_match elements. The first one shows that 295 positions in the read sequence, beginning at position 123, align to 295 positions within the germline gene segments IGHV3-23*01 and IGHV3-23D*01. Alignment of the read sequence to IGHV3-23*01 or IGHV3-23D*01 produces identical results. Thus, they are captured within a single vdj:segment_match element. The resulting alignment has ~93 % sequence identity over the aligned region and 20 nucleotide substitutions. As can be seen in the two vdj:gl_seg_match elements, the two germline gene segments are present in the same germline database (gl_db_id = “1”), they are V gene segments, the alignments were produced using the same alignment software package (aligner_id = “1”), and the alignment begins at the first position of the germline gene segment sequence.

To specify the full alignment for a rearranged gene sequence, we must specify the combination of segment matches inferred to comprise the rearranged sequence. This is captured in the element vdj:combination using the required attribute segments. The value for segments is a list of segment match identifiers pointing to the appropriate vdj:segment_match elements, according to the alignment software package. These are the highest scoring segment matches of each type (V, D, J), arbitrarily using the first one listed when there are multiple segments with equivalent scores. Figure 2 shows the vdj:combination element for the example alignment from Fig. 3. The combination is formed from segment matches with identifiers 1, 3, and 5, corresponding to the germline gene segments IGHV3-23*01 or IGHV3-23D*01, IGHD2-21*01, and IGHJ4*02 (Fig. 4).

### Annotation of alignments

After the full alignment has been specified, it can be annotated with regions of interest using the vdj:region child element of vdj:combination. Regions are specified using the required name, aligner_id, read_pos0, and read_len attributes. The value of name is the name of the region as provided by the software package generating the alignment or annotation. The corresponding software package is indicated using the aligner_id attribute to reference the vdj:aligner element in the vdj:meta section of the VDJML document. read_pos0 and read_len are used to specify precisely where in the read sequence the region is located by specifying the starting location and length, respectively. Commonly annotated regions include framework regions (FRs) 1 – 3, CDRs 1 – 2, the junctions between V and D segments and D and J segments for IGH, TCRB, and TCRD chains, and the junctions between V and J segments for IGK, IGL, TCRA, and TCRG chains. Figure 2 shows example annotations of the VD and DJ junctions and of the FRs and CDRs for an IGH rearrangement.

Alignments can be further annotated using a variety of optional attributes (Table 1). These can be included in either the vdj:segment_match or vdj:region elements.

### VDJML support library

Since the VDJML format is based on XML, existing XML software libraries could be used for VDJML generation, parsing, and validation. However, to simplify use and adoption, we have developed libVDJML – a library for reading, writing, and validating VDJML data – thus eliminating the need to write code for the specific tags and structure of VDJML. By using libVDJML, programs are automatically insulated from changes and enhancements made to the VDJML specification over time. The library is implemented in the C++ language for speed and performance. We currently provide bindings for the Python language and plan support for R and Java.

The library provides the Vdjml_reader and Vdjml_writer classes for reading and writing the contents of a VDJML file. These classes also support compression, so large VDJML files can be compressed using gzip or bzip2, and they do not need to be uncompressed in order to read or write them. As described in the schema above, VDJML consists of a single metadata element (vdj:meta) and any number of alignment results (vdj:read and vdj:segment_match elements). Upon opening a file, Vdjml_reader reads the metadata element, but for efficiency, does not read the VDJML file completely into memory. Instead, it reads only a single vdj:read element at a time. Writing a VDJML file operates in a similar fashion. A metadata entry is provided upon creation of a Vdjml_writer instance, which is written initially to the output file, and alignment results are incrementally serialized.

Each VDJML element and its associated sub-elements for an alignment result are represented by an underlying set of C++ classes, which are instantiated upon parsing of the alignment result. Attributes for VDJML elements are accessed through instance variable getter methods of their respective class in the normal C++ fashion. For elements with variable cardinality, a map data structure is utilized allowing for quick access to an individual element or iteration across all elements. Likewise, construction of elements involves creating the appropriate C++ class with the desired values and inserting any variable cardinality elements; Vdjml_writer will then transform them into a correctly structured VDJML element. All of the capability of the C++ API is available through the Python bindings.

**Fig. 5:**
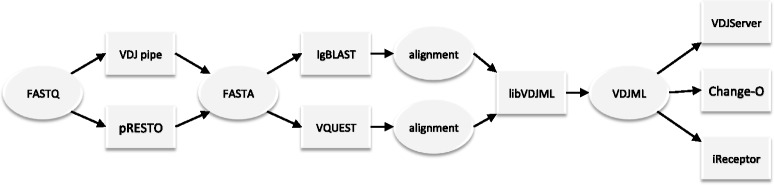
An example workflow showing how libVDJML and VDJML are used with upstream and downstream software packages. *Ovals* indicate file formats, and *rectangles* indicate software packages. This workflow shows how raw sequence read data in FASTQ format is processed to generate FASTA-formatted data for input into germline alignment packages, such as IgBLAST and IMGT/High V-QUEST, which each output their own format. These output files can be read by libVDJML for conversion into VDJML format, which can then be taken as input by a variety of downstream programs

To facilitate conversion of alignment files into VDJML format, libVDJML currently provides the igblast_parse.py script that translates an IgBLAST result file into a VDJML file (Fig. 5). A parser for IMGT/High V-QUEST alignment files is currently under development.

## Results and discussion

We describe VDJML, an XML-based file format designed to represent the results of aligning immune repertoire sequences to germline gene segments. In addition to including sufficient information to reconstruct a full alignment of a sequence to its component germline gene segments, the standard can capture annotations of biological importance, such as CDRs and base substitutions. To our knowledge, this is the first such standard.

We intend for VDJML to be used in at least two ways. First, it is designed to serve as a common format for software developers. If software that generates alignments and their annotations, such as IgBLAST and IMGT/High V-QUEST, generated VDJML files as output, then downstream analysis packages, such as Change-O [23], could be designed to take a single, common file format as input. This would greatly simplify software development by eliminating the need to code against multiple different input formats. Additionally, adoption of VDJML will facilitate the use of multiple different software packages during analysis, eliminating the need for data reformatting. Such standardization is urgently needed. In recent years, there have been significant improvements in high-throughput sequencing of rearranged immune receptor genes, resulting in widespread application of this technology. This has in turn resulted in a tremendous amount of activity developing new software packages to analyze this data type. At this time, we count at least 24 packages, half of which were published in the last two years, and we expect this activity to increase in the coming years.

Our second intended use is as a medium for data sharing. The scientific community at large has recognized the need for data sharing in support of scientific rigor, transparency, and reproducibility. While raw sequence reads, or processed reads used as input to alignment software, can be shared using FASTA/QUAL or FASTQ files, there is benefit to sharing the alignments and annotations used for analysis. It can be complicated and time-consuming to reproduce the full preprocessing, alignment, and annotation steps of an analysis, particularly if one is integrating data from a variety of sources. For some analyses, reproducing all steps may be necessary, but certainly for many it is not. For example, it is frequently of interest to ask whether a particular CDR3 sequence was observed among any donors in a study; this question could be readily addressed with data shared in VDJML format, but would require significant work using FASTA formatted data.

To enhance the transparency of alignments and annotations shared in VDJML format, we have developed elements and attributes for capturing key features of the processes generating a specific VDJML file. In general, the information captured within the vdj:meta section of a VDJML file should enable the file to be recreated if the starting read sequences (and scores if applicable) are available.

Common barriers to the adoption of standards include: (1) a large number of existing, similar standards, (2) not meeting the needs of the target community, and (3) difficulty using the standard. As described above, VDJML is the first file format proposed as a standard for this domain. As such, we don’t anticipate barriers to adoption *if* the format adequately meets the community’s needs and is accompanied by tools to facilitate use. To ensure that the community’s needs are met, VDJML is being developed by a group that includes software developers and non-developer users. Participation is open to all interested. We have an online forum available through http://forums.vdjserver.org. To facilitate use, we have created libVDJML, which includes classes for reading and writing VDJML elements. libVDJML currently supports conversion of IgBLAST output to VDJML, and in the near future, will support conversion from IMGT/High V-QUEST as well.

VDJML is currently supported by software packages developed by authors of this paper. In addition, it will be supported by the ImmPort database [39] as an immune repertoire sequence data reporting format. Currently, for studies that are included in ImmPort, raw immune repertoire sequence data is shared via the Sequence Read Archive (http://www.ncbi.nlm.nih.gov/sra) and VDJML files are shared via a reference to the files hosted at VDJServer. Our test case for developing this system was Rubelt et al. 2016 [40]. The ImmPort study page can be accessed via the study identifier SDY675. The VDJServer study page and files can be accessed here: http://wiki.vdjserver.org/vdjserver/index.php/Rubelt_et_al._2016.

Future development plans for the VDJML format include providing an enhanced representation of workflows within the vdj:meta element. For example, we will include elements to support additional types of processing steps. We will preserve the alignment element and add elements for describing annotations such as genotype corrections and clonal analysis from software such as TIgGER [41] and Change-O [23] for assigning sequences to clones. Additionally, we plan to add elements for acknowledging the probabilistic nature of inferring rearranged sequences and assigning annotations. Future plans for libVDJML include providing bindings to additional languages and expanding the suite of parsers available so that output from existing alignment software can be readily converted to VDJML.

We invite participation from the larger community, both in the form of suggested revisions and enhancements to the schema, as well as in the form of code contributions.
